# Detection of Sensitization Profiles with Cellular In Vitro Tests in Wheat Allergy Dependent on Augmentation Factors (WALDA)

**DOI:** 10.3390/ijms25073574

**Published:** 2024-03-22

**Authors:** Valentina Faihs, Viktoria Schmalhofer, Claudia Kugler, Rebekka K. Bent, Katharina A. Scherf, Barbara Lexhaller, Charlotte G. Mortz, Carsten Bindslev-Jensen, Tilo Biedermann, Per S. Skov, Bernadette Eberlein, Knut Brockow

**Affiliations:** 1 Department of Dermatology and Allergy Biederstein, School of Medicine and Health, Technical University of Munich, 80802 Munich, Germany; 2 Department of Bioactive and Functional Food Chemistry, Institute of Applied Biosciences, Karlsruhe Institute of Technology (KIT), 76131 Karlsruhe, Germany; 3 Odense Research Center for Anaphylaxis (ORCA), Department of Dermatology and Allergy Centre, Odense University Hospital, 5000 Odense, Denmark; 4 RefLab ApS, 2200 Copenhagen, Denmark

**Keywords:** basophil activation test, basophil histamine-release assay, food allergy, gluten, hydrolyzed wheat proteins, IgE, rye, WALDA, WDEIA, wheat allergy dependent on augmentation factors

## Abstract

Wheat allergy dependent on augmentation factors (WALDA) is the most common gluten allergy in adults. IgE-mediated sensitizations are directed towards ω5-gliadin but also to other wheat allergens. The value of the different in vitro cellular tests, namely the basophil activation test (BAT) and the active (aBHRA) and passive basophil histamine-release assays (pBHRA), in the detection of sensitization profiles beyond ω5-gliadin has not been compared. Therefore, 13 patients with challenge-confirmed, ω5-gliadin-positive WALDA and 11 healthy controls were enrolled. Specific IgE (sIgE), skin prick tests, BATs, aBHRA, and pBHRA were performed with allergen test solutions derived from wheat and other cereals, and results were analyzed and compared. This study reveals a distinct and highly individual reactivity of ω5-gliadin-positive WALDA patients to a range of wheat allergens beyond ω5-gliadin in cellular in vitro tests and SPT. In the BAT, for all tested allergens (gluten, high-molecular-weight glutenin subunits, α-amylase/trypsin inhibitors (ATIs), alcohol-free wheat beer, hydrolyzed wheat proteins (HWPs), rye gluten and secalins), basophil activation in patients was significantly higher than in controls (*p* = 0.004–*p* < 0.001). Similarly, significant histamine release was detected in the aBHRA for all test substances, exceeding the cut-off of 10 ng/mL in all tested allergens in 50% of patients. The dependency of tests on sIgE levels against ω5-gliadin differed; in the pBHRA, histamine release to any test substances could only be detected in patients with sIgE against ω5-gliadin ≥ 7.7 kU/L, whereas aBHRA also showed high reactivity in less sensitized patients. In most patients, reactivity to HWPs, ATIs, and rye allergens was observed. Additionally, alcohol-free wheat beer was first described as a promising test substance in ω5-gliadin-positive WALDA. Thus, BAT and aBHRA are valuable tools for the identification of sensitization profiles in WALDA.

## 1. Introduction

Wheat allergy dependent on augmentation factors (WALDA) is an IgE-mediated food allergy, with allergic symptoms occurring when wheat is consumed in combination with augmenting cofactors, such as exercise, nonsteroidal anti-inflammatory drugs, or alcohol [1,2]. Symptom severity in WALDA ranges from isolated urticaria or angioedema to potentially life-threatening anaphylaxis [3,4]. The disease has historically often been labeled as wheat-dependent exercise-induced anaphylaxis (WDEIA) or ω5-gliadin-allergy, but WALDA is the more accurate term [2]. With wheat being the most prevalent food trigger for anaphylaxis in adults in Central Europe [3], the disease is a frequent presentation of food allergy in adults and often leads to more severe reactions than other allergies [3,5].

The diagnosis is made based on the clinical history, the sensitization profile, and an oral challenge test (OCT) with wheat gluten alone and with additional cofactors [1]. The detection of specific IgE (sIgE) to recombinant ω5-gliadin, a relevant allergen in wheat gluten, is an effective screening tool for WALDA, boasting a sensitivity between 80% and 100% [1,6,7]. For skin prick testing (SPT), using pure wheat gluten has delivered the best diagnostic results in WALDA patients [1]. Nonetheless, other gluten allergens such as high-molecular-weight glutenin subunits (HMW-GS), along with potential cross-reactivity to other cereals, may be of relevance for these patients as well [1,8,9,10]. Additionally, recently, nongluten proteins like α-amylase/trypsin inhibitors (ATI) have also been hypothesized to be relevant in WALDA [11].

In contrast to other allergies like Hymenoptera venom allergy [12], the basophil activation test (BAT), based on the measurement of the basophil activation markers such as CD63 by flow cytometry, is not yet a routine diagnostic tool for WALDA. However, the BAT has been shown to discriminate patients and controls and to help identify the individual sensitization patterns to different wheat allergens in WALDA patients, especially when looking at allergens exceeding the established and commercially available extracts [8,11,13]. According to Santos et al., one considerable strength and indication for the BAT is the possibility of testing when no commercial allergen source for sIgE detection is available [14].

Other cellular in vitro tests based on basophil activation include the active and passive basophil histamine-release assays (aBHRA and pBHRA, respectively) [15]. Briefly, in the aBHRA, the amount of histamine released from basophils activated by different test substances is detected. The pBHRA is a slightly modified procedure designed to address the issues of nonreleasing basophils and the need for immediate sample processing [15]. In the pBHRA, the patient’s serum is used to sensitize basophils from a healthy donor from which the autologous IgE has been removed, and then accordingly, the histamine release is quantified upon activation by the allergen of interest [15].

The aBHRA has previously been used in very early studies to investigate the reactivity of WALDA patients to ω5-gliadin and gluten [16,17] and to characterize the sensitization profile in a special cohort of ω5-gliadin-negative WALDA patients sensitized by hydrolyzed wheat proteins (HWPs) in Japan [18,19]. However, to our knowledge, this test has not been used to study sensitization profiles beyond gluten and ω5-gliadin for patients with conventional, ω5-gliadin-positive WALDA, nor has it been used in combination with the BAT and pBHRA.

Therefore, we conducted a study to determine the relevance of different wheat and cereal allergens in patients with WALDA using BAT, aBHRA, and pBHRA, as well as SPT. Besides testing the diagnostic value of wheat beer, the role of known and potential allergens like ATIs and HWPs, as well as cross-reactivity to rye, was assessed for the first time using all three cellular in vitro tests based on basophil activation and SPT.

## 2. Results

### 2.1. Study Population

This study included 13 adult patients with ω5-gliadin-positive, challenge-confirmed WALDA, with a median age of 54 years (range 32–83; 5 females, 8 males) (Supplementary Table S1). The reaction thresholds in the diagnostic OCT with wheat gluten alone and with cofactors are shown in Supplementary Table S1. Additionally, 11 healthy controls without any history of food allergy and tolerance to wheat gluten and cofactors were included (median age 32 years, range 25–67; 6 females, 5 males) (Supplementary Table S1).

### 2.2. Total and Specific IgE

Total IgE levels were found to be higher in the patients than in the healthy controls (median 234 kU/L vs. 60 kU/L, *p* = 0.02; Supplementary Table S1). However, the prevalence of atopic comorbidities was lower in the WALDA patient group (23%) compared with the control group (54.5%; n.s.; Supplementary Table S1). 

As per study inclusion criteria, all patients exhibited sIgE against ω5-gliadin (median 5.4 kU/L, range 2.3–28.4 kU/L), while it was under the detection limit (<0.01 kU/L) in all healthy controls. While significantly higher values were found for sIgE against wheat gluten and gliadins in the patient group (both *p* < 0.001), the difference did not reach the limit for significance for sIgE against wheat (median 0.5 kU/L in the patients vs. 0.1 kU/L in the controls, n.s.) (Supplementary Table S1). Nine out of the thirteen WALDA patients (69%) showed sIgE against rye (>0.35 kU/L). Neither the patients nor the controls showed a sensitization against Tri a 14, the wheat lipid transfer protein (all values < 0.1 kU/L). 

In the patient cohort, sIgE levels against ω5-gliadin showed a significant correlation with sIgE levels against wheat (r = 0.773, *p* = 0.002), gluten (r = 0.775, *p* = 0.002), gliadins (r = 0.890, *p* < 0.001), and rye (r = 0.730, *p* = 0.005).

In the diagnostic OCT, 46% of patients (*n* = 6) showed reactions to artificially high doses of pure wheat gluten alone, not reached in the regular daily diet of the patients, while the diagnosis was confirmed in another 46% (*n* = 6) by adding acetylsalicylic acid and in one patient (7.7%) by additional alcohol and exercise (Supplementary Table S1).

### 2.3. Skin Prick Tests (SPTs)

**Figure 1:**
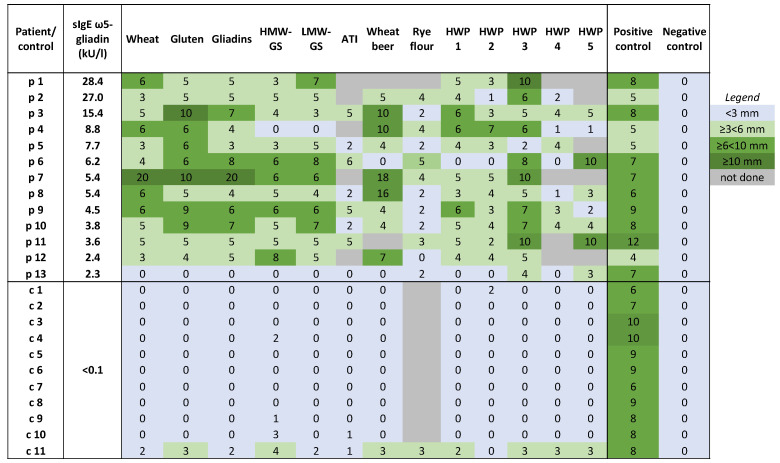
Overview of positive and negative skin prick tests to different allergens in WALDA patients and controls. Data are shown as wheal diameter in mm exceeding the negative control. Histamine dihydrochloride (10%) solution was used as a positive and isotonic sodium chloride solution as a negative control. Abbreviations: ATI, α-amylase/trypsin inhibitors; HMW-GS, high-molecular-weight glutenin subunits; HWPs, hydrolyzed wheat proteins; LMW-GS, low-molecular-weight glutenin subunits; sIgE, specific IgE.

Figure 1 illustrates the results of SPTs in WALDA patients and healthy controls. Significant differences were found in SPT diameters exceeding the negative control between the patients and the controls for all SPT substances. Borderline positive results with some substances were found only in one healthy control (c11) with known atopic comorbidities. 

All but one WALDA patient (p13; 92%) exhibited positive SPT reactions to wheat flour, wheat gluten, and gliadins (median wheal diameters of 5 mm, 6 mm, and 5 mm exceeding the negative control, respectively, vs. 0 mm for all substances in the controls; *p* < 0.001). Eighty-five percent of the patients (11/13) showed positive results for HMW-GS and low-molecular-weight glutenin subunits (LMW-GS), respectively (both median wheal diameter 5 mm, *p* < 0.01 and *p* < 0.001 vs. the controls, respectively). In the SPT, 9/11 (82%) patients showed positive reactions to wheat beer, again with just one borderline positive result in the control subjects (median wheal diameter 5 vs. 0 mm; *p* < 0.001).

Five different HWPs were used for the SPT, and notably, the majority of patients, unlike the controls, showed positive results with the HWPs (HWPs 1,2,3,5: *p* < 0.001 vs. the controls; HWP 4: *p* < 0.01 vs. the controls; median wheal diameters in the patients between 6 mm HWP 3 and 2 mm in HWP 4), yet with very individual response profiles to the different substances, as shown in Figure 1.

No significant correlations could be found for SPT results with sIgE levels against ω5-gliadin in the patients.

### 2.4. Basophil Activation Tests (BATs)

One WALDA patient (p3) was identified as a nonresponder in the BAT due to insufficient response to the positive controls (3.0 and 3.4% CD63^+^ basophils with anti-FcεRI and fMLP, respectively), resulting in exclusion from further analyses. No difference was detected in the background and the positive control values between the patients and healthy controls.

The maximum values of %CD63^+^ basophils (%CD63^+^ max) were significantly higher in the patient group compared with the controls for all allergen test solutions: gluten (median 7.1% vs. 1.6%, *p* < 0.001), HMW-GS (median 10.7% vs. 1.0%, *p* < 0.001), ATIs (median 10.7% vs. 1.6%, *p* < 0.001), alcohol-free wheat beer (median 17.0% vs. 1.2%, *p* < 0.001), extensively hydrolyzed wheat protein (eHWP) (median 6.9% vs. 2.0%, *p* < 0.001), slightly hydrolyzed wheat protein (sHWP) (median 12.3% vs. 1.6%, *p* < 0.001), rye gluten (median 10.8% vs. 1.2%, *p* = 0.002), and rye secalins (median 10.5% vs. 1.2%, *p* = 0.004) (Supplementary Table S2).

**Figure 2:**
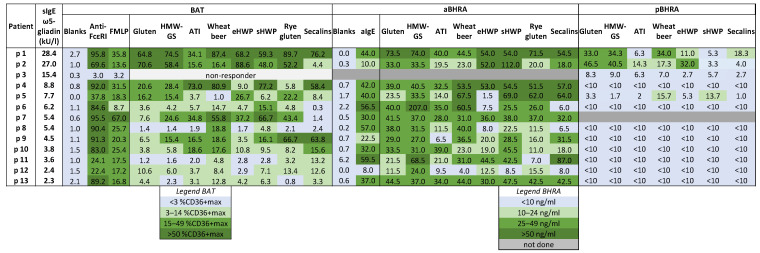
Overview of the in vitro basophil tests, BAT, aBHRA, and pBHRA, in WALDA patients. For the BAT, data are shown as maximum values of % CD63^+^ basophils in any concentration of the respective test substance (%CD63^+^ max). Anti-FcɛRI monoclonal antibodies and N-formyl-methionine-leucyl-phenylalanine (fMLP) were used as positive controls, with two blank determinations as negative controls (mean value shown in the figure). For the aBHRA, the values are presented as maximum histamine release in ng/mL, and anti-IgE (aIgE) was used as the positive control. For the pBHRA, the values are shown as maximum histamine release in ng/mL exceeding the negative control. The color scheme used is purely indicative.

**Figure 3:**
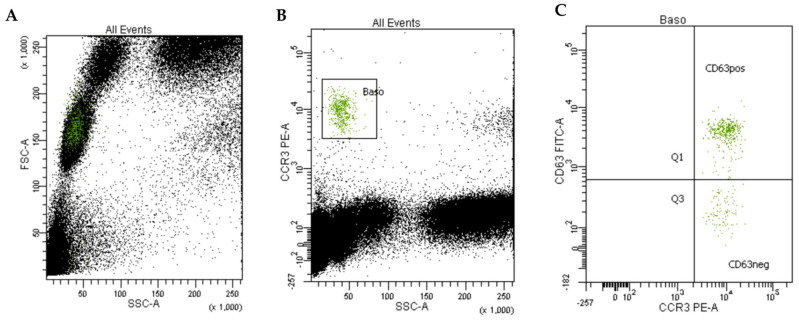
BAT measurement of a WALDA patient (patient 4) with alcohol-free wheat beer in a dilution of 1:100: (**A**) The cells were gated based on their granularity and size using side scatter (SSC-A) and forward scatter (FSC-A). (**B**) Basophil identification was performed using side scatter and the CCR3 identification marker, labeled with an anti-CCR3-phycoerythrin monoclonal antibody. (**C**) The quantification of CD63-positive cells within the total basophil population was performed using CD63 as the basophil activation marker, labeled with anti-CD63-fluorescein-isothiocyanate monoclonal antibodies; in this patient, 81% of 502 counted basophils were CD63^+^ upon stimulation with alcohol-free wheat beer (dilution 1:100).

Figure 2 presents the individual values of the maximum % CD63^+^ basophils for each allergen test solution (%CD63^+^ max) in the included WALDA patients, highlighting the high reactivity observed in most patients to the different test solutions. The results of the 11 healthy controls are shown in Supplementary Figure S1. Surprisingly, the highest rate of activation in the WALDA patients was found for alcohol-free wheat beer in different concentrations. An example of BAT results with alcohol-free wheat beer as a test substance in a concentration of 1:100 is shown in Figure 3.

**Figure 4:**
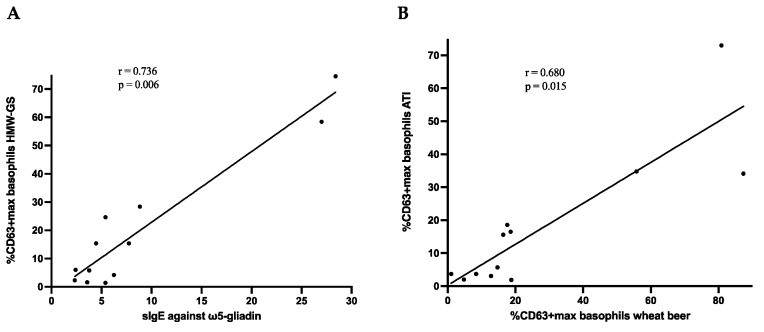
(**A**) Correlation between sIgE against ω5-gliadin and the basophil activation expressed as the maximum %CD63^+^ basophils with HMW-GS (high-molecular-weight glutenin subunits) allergen test solution in the BAT; (**B**) correlation between the basophil activation (expressed as maximum %CD63^+^ basophils) induced by ATIs (α-amylase/trypsin inhibitors) and alcohol-free wheat beer in the basophil activation test.

Generally, patients exhibiting higher sIgE against ω5-gliadin demonstrated increased reactivity to numerous tested extracts (Figure 2). Significant correlations could be found between sIgE levels against ω5-gliadin and reactivity in the BATs with gluten extract (r = 0.650, *p* = 0.02), HMW-GS (r = 0.736, *p* = 0.006; Figure 4A), eHWP (r = 0.706, *p* = 0.01), and sHWP (r = 0.594, *p* = 0.04). However, no correlation was found for alcohol-free wheat beer or rye gluten or secalins, as well as the non-gluten allergen ATIs (all n.s.).

Interestingly, significant correlations could be found in the BAT activation levels (%CD63^+^ max) between ATI, sHWP, and alcohol-free wheat beer (ATI and sHWP: r = 0.932, *p* < 0.001; ATIs and wheat beer: r = 0.680, *p* = 0.015, Figure 4B; sHWP and wheat beer: r = 0.727, *p* = 0.007) in the patients.

In the WALDA patients, the BAT activation levels with eHWP were significantly correlated not only with sIgE levels against ω5-gliadin but also with the % CD63^+^ max with gluten and rye gluten (r = 0.741, *p* = 0.006 and r = 0.643, *p* = 0.02, respectively), as well as with HMW-GS (r = 0.816, *p* = 0.001) and ATIs (r = 0.666, *p* = 0.02).

### 2.5. Active Basophil Histamine-Release Assay (aBHRA)

An overview of the results of the aBHRA in the WALDA patients is presented in Figure 2 and Supplementary Table S2. In line with the BAT findings, a majority of patients demonstrated substantial histamine release to most or all test substances. Notably, 6 out of 12 patients (50%) exhibited a histamine release greater than the cut-off of 10 ng/mL across all allergen test solutions. Similar response patterns to the BATs were observed in several patients; for instance, patients 1, 2, and 4 showed significant responses to all allergen test solutions, while weaker responses were observed for all substances in patient 12, or negative results for patients 6 and 8 with the secalin test substance in all dilutions; however, also exceptions with differing responses can be found for single patients and single allergens.

**Figure 5:**
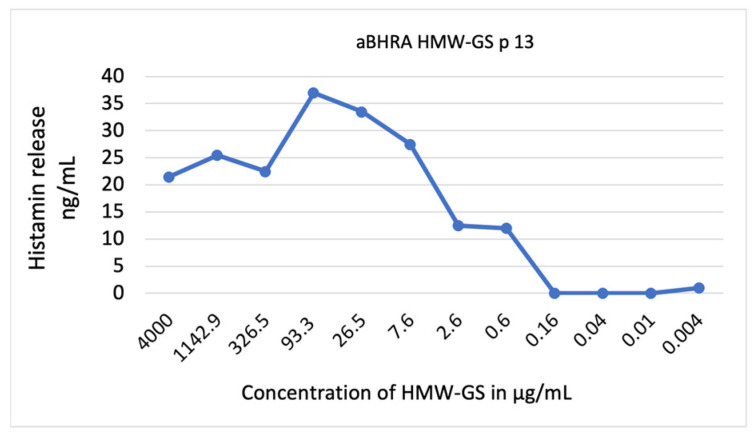
Histamine release (ng/mL) in the aBHRA with HMW-GS (high-molecular-weight glutenin subunits) test solution in different concentrations (in µg/mL) in a patient with WALDA (patient 13).

The patients displayed marked variability in the concentration of allergen test substances that triggered the highest histamine release in the aBHRA, as detailed in Supplementary Table S3. Many patients exhibited a bell-shaped or slope-shaped dose–response curve, typical for aBHRA, with an example illustrated in Figure 5.

Among the various tested concentrations, those leading to the highest median histamine release were often lower than the lowest concentrations used in the BATs, as documented in Supplementary Table S4. For the ATI test solutions, a histamine release over the cut-off of 10 ng/mL could only be detected in patient 4 with an allergen test solution concentration of 0.16 µg/mL, while the responses remained negative with lower concentrations. With rye gluten, just patient 3 showed a histamine release of over 10 ng/mL in the two lowest concentrations (0.01 and 0.004 µg/mL). In all the other used allergen test solutions, responses could still be detected in two or more patients even in the lowest concentrations (Supplementary Table S6).

In contrast to the BATs, no correlation between the histamine release in the aBHRA, except for the secalin test solution and the levels of sIgE against ω5-gliadin, could be found (secalins: r = 0.594, *p* = 0.04; rest *p* > 0.05, n.s.). When looking at the allergen test substance concentration eliciting the maximum response in the aBHRA, a positive correlation with the sIgE against ω5-gliadin could only be found with the ATIs (r = 0.763, *p* = 0.004).

### 2.6. Passive Basophil Histamine-Release Assay (pBHRA)

Figure 2 suggests that in the pBHRA, notable histamine release was limited to the two patients (p1, p2) with the highest sIgE levels against ω5-gliadin. These patients exhibited elevated histamine responses to gluten, HMW-GS, alcohol-free wheat beer, and eHWP, and one of them also exhibited it to ATIs and secalins, as detailed in Figure 2. The concentrations eliciting the highest histamine release are shown in Supplementary Table S5. Another patient (p5), with a medium-level sensitization to ω5-gliadin (7.7 kU/L) displayed marginally elevated histamine responses to wheat beer and sHWP. In contrast, the remaining patients (accordingly, all patients with sIgE against ω5-gliadin below 7.7 kU/L) and one healthy control (c2) (Supplementary Figure S1) did not show notable histamine release in the pBHRA.

### 2.7. Correlations between Applied Tests and Clinical Characteristics

No correlations could be found between the reaction threshold in the OCT with sIgE levels and the maximum CD63^+^ basophils in the BATs or maximum histamine release in aBHRA and pBHRA in the WALDA patients.

A positive correlation was found between the presence of any atopic disease and the sIgE levels against wheat and rye (r = 0.586, *p* = 0.035; and r = 0.685, *p* = 0.01, respectively) but not with the results of BATs, aBHRA or pBHRA.

### 2.8. Responses to Different Test Substance Concentrations in the Cellular In Vitro Tests

As already described, reactivity levels to the different test substance concentrations in all three cellular in vitro tests showed very high interindividual variability. Supplementary Table S6 summarizes the minimum concentrations to obtain positive responses in all three cellular in vitro tests.

## 3. Discussion

Currently, data on the value of cellular in vitro tests to study the sensitization profiles in ω5-gliadin-positive WALDA are limited to BATs [8,11,20]. To our knowledge, for aBHRA and pBHRA, such studies for allergens beyond ω5-gliadin and gluten do not exist. Studies on the BATs showed promising results, especially for assessing patients’ individual sensitization profiles. The first proof-of-principle BATs study by Gabler et al. demonstrated that sensitizations were not only directed against the most prominent gluten allergen, ω5-gliadin but also to HMW-GS and other allergen fractions in WALDA patients [8]. The detection of allergy toward ω5-gliadin is not a diagnostic challenge, as ω5-gliadin sIgE is commonly measured in daily clinical routine as a screening parameter due to the excellent availability of commercial test methods like ImmunoCAP (Phadia, Uppsala, Sweden) [1]. Therefore, in this study, we focused on—partly novel—allergens other than ω5-gliadin to investigate their potential relevance in patients with ω5-gliadin-positive WALDA using the cellular in vitro tests aBHRA and pBHRA in addition to the more established BATs.

Our study reveals a distinct and highly individual reactivity of ω5-gliadin-positive WALDA patients to a range of wheat allergens beyond ω5-gliadin, including gluten proteins, HWPs, and water-soluble, nongluten proteins like ATIs, in different basophil in vitro tests and in SPTs. Reactivity against different allergens in ω5-gliadin-positive WALDA patients has already been reported in some studies [8,21,22]. It is still unclear whether this represents a cross-reactivity between epitopes of ω5-gliadin and other proteins or a different primary clinically relevant cosensitization against other allergens in some patients. The diverse and complex immune responses of WALDA patients to various allergens are the main limitations for the development of hypoallergenic wheat [9,10]. Thus, Morita et al. recently emphasized the necessity for improved diagnostic methods to pinpoint the patients’ individual sensitization profiles beyond ω5-gliadin [10].

To our knowledge, the only study comparing the BATs with the aBHRA and pBHRA in food allergy was performed by Larsen et al. in peanut-allergic subjects, showing good results in all of three tests [15]. Despite being important cellular allergy tests, the aBHRA and pBHRA have not been used to study the sensitization profiles in ω5-gliadin-positive patients beyond the allergens ω5-gliadin and gluten. The most relevant advantages of cellular in vitro basophil tests in comparison with sIgE are that (1) they are able to demonstrate biological basophil reactivity to different allergens in addition to allergen binding and that (2) multiple and novel test extracts can be tested when compared with sIgE detection, which is only available for a few established allergens (for example by ImmunoCAP). In this study, substantially more allergen concentrations were tested by automated histamine level detection in the BHRA, as the manual analysis of different dilutions of allergen test solutions in the BATs is far more time-consuming. This is an advantage, especially in exploratory studies and when using new allergen extracts. While we based the initial BAT concentrations on previous studies [8,11] for the previously tested substances and on dose-finding experiments with healthy controls for the newly used substances, the BHRAs were able to unravel the patients’ extremely variable optimum, eliciting concentrations with far more used allergen concentrations.

The previously described utility of the BATs in identifying patients’ individual sensitization profiles could be confirmed in this study [8,11,20]. Additionally, the aBHRA yielded encouraging results in identifying the patients’ sensitization profiles and optimal concentrations. This is not surprising, as the methodology is quite similar to the BATs except for the quantification method [14]. Interestingly, the BATs showed a stronger dependence of reactivity to allergens on higher sIgE levels against ω5-gliadin compared with aBHRA, while still working in weakly sensitized subjects. This finding should be validated in a larger number of patients. Of note, in this study, the patient with the lowest sIgE levels against ω5-gliadin (p13) showed negative SPT results to all but one substance and low activation levels in the BATs but strong responses in the aBHRA. The pBHRA showed the strongest dependence of reactivity on high ω5-gliadin sIgE levels and resulted in measurable histamine release to any test substance just in three patients with sIgE against ω5-gliadin ≥ 7.7 kU/L. This aligns with previous pBHRA studies [23] and the study by Larsen et al., where most included patients displayed high sIgE levels to peanut allergens, and the pBHRA failed in two patients with just moderate sensitization [15]. This led the authors to speculate that pBHRA might pose diagnostic challenges in patients with moderate-to-low sIgE levels—consequently, they suggested using the pBHRA as a secondary, confirmatory test following BATs or aBHRA, rather than as a standalone diagnostic tool [15]. Additionally, the pBHRA represents an alternative if BATs or BHRA cannot be performed due to the lack of fresh full blood. Taken together, in this study, we could confirm the previously reported [12,14,15,24] strengths and limitations of the three different in vitro tests; all of them are useful when no commercial allergen source for sIgE detection is available, and they represent good models for basophil reactivity. The BATs and aBHRA have the limitation of immediate sample processing and some patients having nonresponding basophils, but they show good results in weakly sensitized patients. On the contrary, the pBHRA can easily be performed with patients’ frozen serum, but it shows a high dependency on sIgE levels. The allergen doses resulting in maximum responses in the cellular tests showed a very high intraindividual reactivity, so testing with a wide range of concentrations is suggested for future research.

The results of this study show that most ω5-gliadin-positive WALDA patients had positive results in the SPTs and in vitro basophil tests to HMW-GS, confirming them as major allergens in WALDA [8,25]. Unfortunately, to date, sIgE to HMW-GS cannot be detected commercially. Cellular tests are necessary to identify sensitization towards HMW-GS. However, test substances for HMW-GS are not widely available, and the production of the test substance used in this study requires elaborate production processes. 

In contrast, wheat beer is easily accessible. Surprisingly, wheat beer (alcohol-free) showed excellent results as an allergen test substance in SPTs, as well as in BAT and aBHRA, with positive results in most WALDA patients and negative results in most of the controls. In Germany, wheat beer is produced with a mandatory minimum of 50% wheat malt [26]. It has been shown that in German wheat beer, several ATIs can be detected, along with gliadins, glutenins, lipid transfer proteins, and serpins, and that the gluten proteins show different degrees of hydrolysis due to the production process [26,27,28]. Interestingly, we could find significant correlations between the BAT activation levels with alcohol-free wheat beer and ATIs, as well as sHWP (primarily consisting of ATIs [11]), while this was not the case for any wheat gluten allergens or sIgE to ω5-gliadin. The clinical relevance of this finding is unclear, as there have been few reported cases of anaphylaxis to wheat beer to date [29,30,31]. Currently, reactivity to wheat beer seems to be a promising biomarker for WALDA. Further studies are needed to confirm the utility of wheat beer as a test substance, as well as the clinical tolerance in WALDA patients.

Wheat ATIs, water-soluble proteins involved in plant defense, have been shown to play a role in bakers’ asthma [32,33]. It is still unclear whether they are relevant allergens in WALDA. This possibility was suggested by an early study by Pastorello et al. [34] and further supported by Gabler et al. [11], who found that saline gluten extracts, primarily containing ATIs, triggered positive BAT responses in WALDA patients but not in controls. We could confirm their results in our study, with positive responses seen for most patients in the SPTs, as well as in the BATs and aBHRA. In the Gabler et al. study [11], the same eHWP and sHWP extracts as those used in our study were used and analyzed. ATIs were found to predominate in the sHWP solution, whereas gliadins and glutenins were the major fractions in eHWP. Accordingly, in this study, we could find significant correlations between the BAT activation levels of sHWP and ATIs, whereas BAT results with eHWP showed a correlation to those with gluten and HMW-GS, as well as with sIgE levels against gliadins and ω5-gliadin. 

Additionally, in this study, various HWPs were used as test substances. HWPs, derived from gluten through chemical or enzymatic partial hydrolysis, exhibit increased solubility, as well as enhanced foaming and emulsifying abilities, making them frequently used in cosmetic products and foods [35]. Their functional properties and molecular composition vary depending on the treatment method [35,36], and hydrolysis may reveal or create new allergenic epitopes or influence allergen absorption [18,37,38]. HWPs in cosmetics are recognized as a potential cause of percutaneous sensitization leading to WALDA [19,37,39]. However, patients sensitized through HWPs typically lack sIgE against ω5-gliadin, thus distinguishing them from our study’s cohort [37]. In this study, a significant proportion of “classical”, ω5-gliadin-positive WALDA patients showed a sensitization to different HWP, while this could not be observed in the healthy controls. To our knowledge, this is the first report to show that a majority of ω5-gliadin-positive WALDA patients, but not the healthy controls, show positive results with different HWPs in the SPTs, yet with very individual and selective responses based on the different HWPs. A possible explanation could be that various new epitopes become apparent based on the treatment method of each product, eliciting individual reactions in different patients based on their individual immunological patterns. Thus, HWPs appear to play an important role not only in ω5-gliadin-negative WALDA but also in the classical, ω5-gliadin-positive subtype of the disease—however, the clinical relevance still has to be clarified. 

Interestingly, more than half of ω5-gliadin-positive WALDA patients showed sensitization to rye allergens in the different applied tests (sIgE, SPTs, BATs, aBHRA). These findings are consistent with a study by Kennard et al., which reported sIgE to rye in 77% of WALDA patients [40]. In vitro studies suggest a cross-reactivity between the ω5-gliadin in wheat and γ-secalins found in rye [41,42]. Accordingly, the majority of patients also showed sensitization to rye secalins in the BAT and aBHRA. To our knowledge, this is the first study to assess the reactivity to rye allergens in ω5-gliadin-positive WALDA by cellular in vitro tests. However, to our knowledge, no study on the clinical tolerance of rye in WALDA patients has been published yet.

Finally, certain limitations in our study have to be acknowledged. First, the number of patients is limited. Nevertheless, it is important to note that WALDA patient populations are generally small in most healthcare centers and research studies, especially when diagnosed by OCT, and that the BATs and aBHRA must be performed with fresh blood, leading to organizational effort. Second, this study focused specifically on the classical subtype of ω5-gliadin-positive WALDA, so it cannot draw any conclusions about patients with ω5-gliadin-negative WALDA. Third, due to the exploratory and proof-of-principle character of this initial study, no experiments were performed in healthy controls for the aBHRA, and the maximum concentrations were thus adopted from the BAT experiments. With the knowledge of the BHRA experiments, testing for more and even lower concentrations in the BATs might have delivered valuable insights and more detailed information about the individual dose–response curves.

Despite these limitations, our initial results lay the basis for further research on relevant allergens involved in WALDA and the application of different in vitro basophil tests in this patient collective.

## 4. Materials and Methods

### 4.1. Study Population

From September 2021 to July 2023, 13 patients diagnosed with challenge-confirmed WALDA were enrolled in the study. Inclusion criteria comprised a history of allergic reactions following wheat consumption with augmentation factors, despite a normal tolerance for wheat products alone. Additional criteria included sIgE levels to ω5-gliadin ≥ 0.35 kU/L (ImmunoCAP assay, Phadia, Uppsala, Sweden), a positive oral challenge test (OCT) to wheat gluten alone or with augmentation factors, and the availability of results of at least two molecular in vitro tests (BAT, aBHRA, pBHRA). Additionally, 11 healthy controls without any history of food allergy, with tolerance to wheat gluten alone and cofactors and nondetectable sIgE to ω5-gliadin (<0.1 kU/L) (ImmunoCAP, Phadia, Uppsala, Sweden), were recruited as volunteers. This study received approval from the ethics committee of the Technical University of Munich (approval number 477/21 S-NP), and all participants provided written informed consent before their inclusion in this study.

### 4.2. Clinical History, IgE Diagnostics, Skin Prick Tests, and Oral Challenge Tests

Each patient underwent a thorough evaluation of their medical history, secondary diagnoses, and atopic comorbidities. 

Blood samples were collected to assess total IgE and sIgE levels to wheat flour (f4), gluten (f79), gliadin (f98), the wheat lipid transfer protein Tri a 14 (f433), ω5-gliadin (Tri a 19, f416), and rye (g5), as well as basal serum tryptase (ImmunoCAP assay, Phadia, Uppsala, Sweden). 

Skin prick tests (SPTs) were performed as prick-to-prick tests using the following substances: native wheat flour (type 550); wheat gluten; rye flour (all commercially available); wheat beer (Paulaner Brauerei, Munich, Germany); five hydrolyzed wheat proteins (HWPs 1–5), which different manufacturers kindly provided; isolated LMW-GS, HMW-GS, and gliadins (produced as previously described [8]); and α-amylase/trypsin inhibitors (ATI; Merck KGaA, Darmstadt, Germany; 5–10 mg/mL dilution in isotonic saline solution). Histamine dihydrochloride (10 mg/mL; ALK-Abello, Copenhagen, Denmark) was used as the positive control solution, and 0.9% saline was used as the negative control, respectively. Results with a mean wheal diameter of 3 mm or greater compared with the negative control after 20 min were considered positive. 

The oral challenge tests (OCTs) were conducted in an inpatient setting for the patients and in an outpatient setting for the controls, adhering to a previously published protocol [1]. Reaction thresholds were evaluated using an ordinal scale from 1 to 10, as previously published [43].

### 4.3. Allergen Test Solutions for In Vitro Tests

The in vitro test solutions for wheat gluten, gliadins, and HMW-GS were produced as previously described in [8] by pepsin hydrolysis to increase water solubility. Additionally, saline extracts of eHWP and sHWP were produced by aqueous extraction, as described in [11], where these extracts were also extensively characterized. Rye secalin extract was produced by pepsin hydrolysis of resolubilized rye prolamins from rye flour prepared by Osborne fractionation [44]. Rye gluten extract was produced by pepsin hydrolysis of resolubilized rye gluten extracted by the same procedure but directly with a salt solution, followed by the glutelin solution without the extraction step using 60% ethanol [44].

α-amylase/trypsin inhibitors were from Merck KGaA (Darmstadt, Germany). Alcohol-free wheat beer from Paulaner Brauerei (Munich, Germany) was used for the wheat beer allergen test solution. After initial dose titration experiments, the allergen test solution concentrations shown in Table 1 were selected for the BAT, as they did not lead to unspecific stimulation in healthy controls. Thus, the upper concentration limits were applied to the aBHRA and pBHRA too, and only concentrations equal to or lower than those used in the BATs were used, as presented in Table 1. More dilution steps were tested in BHRA than in BAT due to the time-optimized procedure and in order to obtain better dose–response curves. The steps of dilution were chosen in an exploratory manner due to previous lab standards.

### 4.4. Basophil Activation Tests (BATs)

The Flow CAST method (Buehlmann Laboratories AG, Schoenenbuch, Switzerland) was used as previously described [8,11]. Briefly, 9 mL venous blood was collected in EDTA tubes and gently homogenized at room temperature. For each measurement, 50 μL of allergen test solution (in different concentrations, see Table 1; diluted in stimulation buffer from Buehlmann Laboratories AG, Schoenenbuch, Switzerland), 100 μL of stimulation buffer, 50 μL of blood, and 20 μL of staining reagent were gently mixed in polystyrene tubes. The staining reagent consisted of anti-CD63-fluorescein-isothiocyanate and anti-CCR3-pycoerythrin monoclonal antibodies. The tubes were then incubated for 25 min at 37 °C. By adding 2 mL lysis reagent and standing for 5 min in the dark at room temperature, the stimulation was stopped. After 5 min of centrifugation, the supernatant was decanted, and the cell pellet was resuspended in 300 μL of wash buffer. Anti-FcεRI monoclonal antibodies and N-formyl-methionyl-leucyl-phenylalanine (fMLP) were used as positive controls. Stimulation buffer alone was used to determine the background value in two separate measurements. The flow cytometric analysis was conducted using the flow cytometer BD FACSCanto II with 488 nm excitation wavelength (argon ion laser) and the BD FACSDiva Software (version 9.0; Becton-Dickinson Biosciences GmbH, Heidelberg, Germany). Basophils were gated as low side scatter CCR3/side scatter^low^. CCR3 was used to identify basophils and CD63 as basophil activation marker both marked with fluorescence-dye-labeled monoclonal antibodies. Each measurement included the counting of ≥450 basophils. The percentage of activated basophils (%CD63^+^ basophils) was determined by calculating the proportion of basophils expressing CD63 relative to the overall count of basophils in each measurement, according to previous studies [8].

### 4.5. Active Basophil Histamine-Release Assay (aBHRA)

The active basophil histamine-release assay (aBHRA) was performed as previously described [15]. In brief, heparinized whole blood was collected and after centrifugation, and the resulting plasma was substituted with 1,4-piperazinediethanesulfonic acid (PIPES) buffer (RefLab, Copenhagen, Denmark). Glass fiber-coated microtiter plates from RefLab (Copenhagen, Denmark) were used for the subsequent steps. All determinations were performed in duplicates to calculate mean values. In each well, 50 μL of blood and 50 μL of allergen test solution in different concentrations (see Table 1) were combined and incubated at 37 °C for 60 min. After repeated washing with demineralized water, the plates were incubated with 0.4% wash buffer (0.4% sodium dodecyl sulfate, RefLab) for 10 min. After repeated washing, the plates were incubated with 0.1 mg/mL o-phthaldialdehyde (RefLab) at alkaline pH. After 10 min, the reaction was halted by lowering the pH with 0.59% perchloric reagent (RefLab), and the fluorescence intensity of each well was measured using a Histareader from RefLab. The obtained fluorescence intensity values were then converted to amounts of released histamine in ng/mL using a standard curve.

### 4.6. Passive Basophil Histamine-Release Assay (pBHRA)

Donor basophils obtained from blood bank buffy coats were selected and screened as previously described [23,45]. Samples of 500 µL IgE-stripped buffy coat basophils were passively sensitized by incubation with 125 µL serum from WEDIA patients. Thereafter, the passively sensitized blood samples were resuspended with Pipes buffer to a total volume of 2.5 mL. Cell suspension aliquots of 25 µL were challenged by incubation at 37 °C for 60 min with 25 µL of the allergens (see Table 1) in 12 concentrations with 3.5-fold dilution steps. After incubation, histamine was quantitated as described above for aBHRA. The histamine release induced by the different allergen test solutions was calculated as the histamine release exceeding the negative control.

### 4.7. Statistics

Statistical Package for Social Sciences Statistics 29.0 (SPSS Inc., Chicago, IL, USA), Microsoft Excel (version 16.83; Microsoft Corporation, Redmond, WA, USA), and GraphPad PRISM version 10 (GraphPad Software Inc., La Jolla, CA, USA) were used for statistical analyses and data visualization. Patient characteristics are described as median and range for continuous variables and as frequency and proportion for dichotomous variables. The maximum values for % CD63^+^ basophils (%CD63^+^ max) out of all tested concentrations of each allergen test solution in patients and controls in the BAT (based on [8]) and the maximum histamine release (in ng/mL) for the aBHRA and the and the maximum histamine release in ng/mL exceeding the negative control for the pBHRA were used to calculate differences and correlations. After using the Shapiro–Wilk test to assess the normality of data distribution, statistical analyses for non-normally distributed data were used. Differences between groups were evaluated using the Mann–Whitney U-test, and Spearman’s rank correlation coefficients were utilized to determine correlations. A *p*-value of <0.05 was considered statistically significant.

## 5. Conclusions

This study highlights the complex and individual nature of molecular allergen reactivity beyond ω5-gliadin in ω5-gliadin-positive WALDA patients. In this study, the BATs were confirmed to be an effective tool for identifying patients’ individual sensitization profiles with noncommercially available allergen test solutions compared with healthy controls. Significantly basophil activation levels were found in patients compared with controls for gluten, HMW-GS, ATI, wheat beer eHWP, and sHWP (all *p* < 0.001), as well as rye gluten and secalins (*p* = 0.002 and 0.004, respectively). This is the first study to apply the aBHRA to assess the sensitization profiles in ω5-gliadin-positive WALDA patients beyond the allergens ω5-gliadin and gluten, and encouraging results could be observed, with strong levels of histamine release observed with all test substances. In contrast, the pBHRA was only useful in patients highly sensitized against ω5-gliadin (≥7.7 kU/L), which is in line with previous studies showing a high dependency on sIgE levels. This is the first study evaluating wheat beer as a promising allergen test substance in WALDA, opening new perspectives for easily accessible test substances. Additionally, ATIs and HWPs have been identified as potentially relevant allergens in ω5-gliadin-positive WALDA, underscoring the need for further research on their clinical relevance. However, further research is needed to confirm these findings with larger numbers of patients and healthy, age-matched controls.

## Figures and Tables

**Table 1:** Concentrations of the tested allergens in BATs and BHRA.

Allergen Test Solution	Concentrations in BATs	Concentrations in aBHRA	Concentrations in pBHRA
Gluten	4000/2000/800 µg/mL	4000/1142.9/326.5/93.3/26.5/7.6/2.6/0.6/0.16/0.04/0.01/0.004 µg/mL	3600/1028/293/84/24/6.9/0.2/0.6/0.2/0.05/0.01/0.004 µg/mL
HMW-GS	4000/2000/800 µg/mL	4000/1142.9/326.5/93.3/26.5/7.6/2.6/0.6/0.16/0.04/0.01/0.004 µg/mL	3600/1028/293/84/24/6.9/0.2/0.6/0.2/0.05/0.01/0.004 µg/mL
ATIs	400/200/80/40 µg/mL	326.5/93.3/26.5/7.6/2.6/0.6/0.16/0.04/0.01/0.004 µg/mL	293/84/24/6.9/0.2/0.6/0.2/0.05/0.01/0.004 µg/mL
Alcohol-free wheat beer	1:10/1:100	1:12.2/1:42.9/1:150.1/1:525.2/1:1838.8/1:6430.7/1:22,517.4/1:78,710.9/1:275,487.5/1:964,706.8	1:12.2/1:42.9/1:150.1/1:525.2/1:1838.8/1:6430.7/1:22,517.4/1:78,710.9/1:275,487.5/1:964,706.8
eHWP	1:5/1:10/1:50	1:12.2/1:42.9/1:150.1/1:525.2/1:1838.8/1:6430.7/1:22,517.4/1:78,710.9/1:275,487.5/1:964,706.8	1:12.2/1:42.9/1:150.1/1:525.2/1:1838.8/1:6430.7/1:22,517.4/1:78,710.9/1:275,487.5/1:964,706.8
sHWP	1:5/1:10/1:50	1:12.2/1:42.9/1:150.1/1:525.2/1:1838.8/1:6430.7/1:22,517.4/1:78,710.9/1:275,487.5/1:964,706.8	1:12.2/1:42.9/1:150.1/1:525.2/1:1838.8/1:6430.7/1:22,517.4/1:78,710.9/1:275,487.5/1:964,706.8
Rye gluten	4000/2000/800 µg/mL	4000/1142.9/326.5/93.3/26.5/7.6/2.6/0.6/0.16/0.04/0.01/0.004 µg/mL	not tested
Rye secalins	800 µg/mL	26.5/7.6/2.6/0.6/0.16/0.04/0.01/0.004 µg/mL	24/6.9/0.2/0.6/0.2/0.05/0.01/0.004 µg/mL

The concentration values indicate the concentration of the allergen test substances after reconstitution. Abbreviations: ATI: α-amylase/trypsin inhibitors. HMW-GS: high-molecular-weight glutenin subunits. eHWP: extensively treated hydrolyzed wheat proteins. sHWP: slightly treated hydrolyzed wheat proteins.

## Data Availability

The data that support the findings of this study are available from the corresponding author upon reasonable request.

## Supplementary Materials

The following supporting information can be downloaded at https://www.mdpi.com/article/10.3390/ijms25073574/s1.
