# Supplementary material for: Detection of Sensitization Profiles with Cellular In Vitro Tests in Wheat Allergy Dependent on Augmentation Factors (WALDA)
Source: Int J Mol Sci. 2024 Mar 22;25(7):3574. doi: 10.3390/ijms25073574 (PMC11012217; doi:10.3390/ijms25073574)
Supplement: Supplementary file 1 [file ijms-25-03574-s001.zip › ijms-2894005-supplementary.pdf]

International Journal of
Molecular Sciences

MDPI

Detection of Sensitization Profiles with Cellular In Vitro Tests in Wheat Allergy Dependent on Augmentation Factors (WALDA)

Valentina Faihs [1], Viktoria Schmalhofer [1], Claudia Kugler [1], Rebekka K. Bent [1], Katharina A. Scherf [2], Barbara Lexhaller [2], Charlotte G. Mortz [3], Carsten Bindslev-Jensen [3], Tilo Biedermann [1], Per S. Skov [3,4], Bernadette Eberlein [1] and Knut Brockow [1,3,*]

[1] Department of Dermatology and Allergy Biederstein, School of Medicine and Health, Technical University of Munich, 80802 Munich, Germany
[2] Department of Bioactive and Functional Food Chemistry, Institute of Applied Biosciences, Karlsruhe Institute of Technology (KIT), 76131 Karlsruhe, Germany
[3] Odense Research Center for Anaphylaxis (ORCA), Department of Dermatology and Allergy Centre, Odense University Hospital, 5000 Odense, Denmark
[4] RefLab ApS, 2200 Copenhagen, Denmark
* Correspondence: knut.brockow@tum.de; Tel.: +49-894140-3069; Fax: +49-894140-3576

Supplementary material

Table S1. Clinical characteristics of WALDA patients and healthy controls.

Clinical characteristics	WALDA patients, n=13	Controls, n=11	p-value
Age, median (range)	54 years (32-83)	32 years (25-67)	<0.05
Sex, male/female, n (%)	8/5 (61.5%/38.5%)	5/6 (54.5%/45.5%)	n.s.
Any self-reported atopic comorbidities, n (%)	3 (23%)	6 (54.5%)	n.s.
Total IgE, median (range)	234 kU/l (52.4-2848)	59.7 kU/l (3.69-488)	<0.05
sIgE to ω5-gliadin, median (range)	5.40 kU/l (2.32-28.4)	<0.1 kU/l	<0.001
sIgE to wheat, median (range)	0.51 kU/l (<0.1-4.65)	0.12 kU/l (<0.1-1.19)	n.s.
sIgE to wheat gluten, median (range)	1.77 kU/l (0.13-9.95)	<0.1 kU/l (<0.1-0.3)	<0.001
sIgE to gliadins, median (range)	1.44 kU/l (<0.1-10.3)	<0.1 kU/l	<0.001
sIgE to rTri a 14 wheat, median (range)	<0.1 kU/l (<0.1-0.12)	<0.1 kU/l	n.s
Positive SPT with wheat flour, n (%)	12 (92.3%)	0 (0%)	<0.001
Positive SPT with wheat gluten, n (%)	12 (92.3%)	1 (0.9%)	<0.001
Basal serum tryptase, median (range)	4.31 µg/l (3.01-6.66)	2.75 µg/l (0.4-11.7)	n.s
Oral challenge test reaction threshold*, n (%)	1) 3 (23%)<br>2) 0 (0%)<br>3) 3 (23%)<br>4) 3 (23%)<br>5) 2 (15.4%)<br>6) 1 (7.7%)<br>7) 0 (0%)<br>8) 1 (7.7%)<br>9) 0 (0%)<br>10) 0 (0%)	-	-

* see Methods section for grading. Abbreviations: sIgE, specific IgE; SPT, skin prick test.

Table S2. Overview of the results of cellular in vitro tests in WALDA patients and in healthy controls.

In vitro test	Test substance	WALDA patients	Controls	p-value
**BAT**<br>%CD63+max in any concentration, median (range)	Gluten	7.1 (1.2-64.8)	1.6 (0.4-3.7)	<0.001
	HMW-GS	10.7 (1.4-74.5)	1.0 (0.4-7.9)	<0.001
	ATI	10.7 (1.9-73.0)	1.6 (0.6-2.7)	<0.001
	Wheat beer	17.0 (1.0-87.4)	1.2 (0.2-22.5)	<0.001
	eHWP	6.9 (1.7-88.6)	2.0 (1.0-3.6)	<0.001
	sHWP	12.3 (2.8-77.2)	1.6 (0.2-7.4)	<0.001
	Rye gluten	10.8 (0.8-89.7)	1.2 (0.6-6.2)	0.002
	Rye secalins	10.5 (0.3-76.2)	1.2 (0.4-8.8)	0.004
**aBHRA**<br>maximum histamine release (ng/ml) in any concentration, median (range)	Gluten	35.8 (11.5-73.5)	n.d.	
	HMW-GS	35.3 (24.0-207.0)	n.d.	
	ATI	24.5 (6.5-40.0)	n.d.	
	Wheat beer	38.3 (4.0-67.5)	n.d.	
	eHWP	25.0 (1.5-54.0)	n.d.	
	sHWP	44.0 (8.5-112.0)	n.d.	
	Rye gluten	23.0 (7.0-71.5)	n.d.	
	Rye secalins	31.8 (6.0-87.0)	n.d.	
**pBHRA**<br>maximum histamine release (ng/ml exceeding the negative control) in any concentration, median (range)	Gluten	<10.0 (3.3-46.5)	5.7	
	HMW-GS	<10.0 (1.7-40.5)	5.0	
	ATI	<10.0 (2.0-14.3)	2.3	
	Wheat beer	<10.0 (7.0-34.0)	2.7	
	eHWP	<10.0 (2.7-32.0)	2.3	
	sHWP	<10.0 (5.3-33.3)	2.0	
	Rye secalins	<10.0 (1.0-18.3)	0.0	

Abbreviations: BAT, basophil activation test; aBHRA, active basophil histamine release assay; pBHRA, passive basophil histamine release assay; ATI, α-amylase/trypsin inhibitors; HMW-GS, high-molecular-weight glutenin subunits; eHWP, extensively hydrolyzed wheat proteins; sHWP, slightly hydrolyzed wheat proteins.

Control subject	sIgE ω5-gliadin (kU/l)	BAT											pBHRA						
		Blanks	Anti-FcεRI	FMLP	Gluten	HMW-GS	ATI	Wheat beer	eHWP	sHWP	Rye gluten	Secalins	Gluten	HMW-GS	ATI	Wheat Beer	eHWP	sHWP	Secalins
c 1		0.2	79.2	42.2	0.4	0.8	0.6	0.8	1.6	0.2	0.6	1.2							
c 2		0.9	84.4	38.0	0.6	0.8	1.4	1.4	2.2	2.9			5.7	5.0	2.3	2.7	2.3	2.0	0.0
c 3		0.4	19.4	16.2	0.6	0.4	1.6	0.2	1.0	0.4	0.6	2.4							
c 4		**0.8**	**85.6**	**23.2**	**1.8**	**1.2**	**2.0**	**1.2**	**3.4**	**1.4**	**1.0**	**1.4**							
c 5		**1.4**	**62.0**	**17.2**	**3.4**	**2.2**	**1.2**	**2.4**	**3.6**	**7.4**	**6.2**	**1.7**							
c 6	<0.1	**1.3**	**92.3**	**4.7**	**0.6**	**0.8**	**2.6**	**2.2**	**1.2**	**1.4**	**1.6**	**1.2**							
c 7		**0.8**	**53.2**	**13.6**	**2.4**	**1.0**	**2.1**	**2.5**	**2.1**	**0.6**	**1.2**	**0.6**							
c 8		**1.0**	**82.1**	**70.3**	**3.7**	**0.5**	**1.1**	**0.9**	**1.2**	**4.2**	**1.0**	**0.6**							
c 9		**0.4**	**86.8**	**10.8**	**1.7**	**7.9**	**2.7**	**22.5**	**1.4**	**1.7**	**3.4**	**0.6**							
c 10		**0.4**	**40.7**	**44.6**	**1.0**	**1.8**	**2.6**	**0.4**	**2.4**	**2.2**	**1.2**	**0.4**							
c 11		**1.5**	**52.0**	**13.5**	**1.6**	**4.0**	**1.4**	**1.2**	**2.0**	**1.6**	**1.6**	**8.8**							

Legend BAT
<3 %CD36+max
3-14 %CD36+max
15-49 %CD36+max
>50 %CD36+max
not done

Legend pBHRA
<10 ng/ml
10-24 ng/ml
25-49 ng/ml
>50 ng/ml
not done

Figure S1. Overview of the *in vitro* basophil tests BAT and pBHRA in healthy controls. For the BAT, data are shown as maximum values of %CD63+ basophils in any concentration of the respective test substance (%CD63+max). Anti-FcεRI monoclonal antibodies

and N-formyl-methionine-leucyl-phenylalanine (fMLP) were used as positive controls, two blank determinations as negative controls (mean value shown in figure). For the pBHRA, the values are shown as histamine release in ng/ml exceeding the negative control. The colour scheme used is purely indicative.

Table S3. Concentrations of the allergen test solutions in µg/ml eliciting the highest histamine release in the aBHRA in WALDA patients.

Patient	sIgE against ω5-gliadin (kU/l)	Gluten	HMW-GS	ATI	Alcohol-free wheat beer	eHWP	sHWP	Rye gluten	Rye secalins
**p 1**	28.4	4000	4000	326.5	1:6430.7	1:12.2	1:12.2	4000	0.04
**p 2**	27.0	4000	4000	326.5	1:12.2	1:525.2	1:275487.5	1142.9	26.5
**p 3**	15.4	2.6	0.6	326.5	1:12.2	1:78710.9	1:275487.5	0.6	0.04
**p 4**	8.8	not done							
**p 5**	7.7	26.5	326.5	326.5	1:6430.7	1:78710.9	1:1838.8	1142.9	2.6
**p 6**	6.2	26.5	26.5	326.5	1:42.9	1:12.2	1:6430.7	93.3	26.5
**p 7**	5.4	26.5	1142.9	326.5	1:42.9	1:22517.4	1:12.2	1142.9	26.5
**p 8**	5.4	0.16	4000	326.5	1:12.2	1:6430.7	1:12.2	7.6	0.16
**p 9**	4.5	4000	4000	93.3	1:42.9	1:12.2	1:12.2	93.3	7.6
**p 10**	3.8	4000	4000	326.5	1:42.9	1:12.2	1:12.2	4000	0.16
**p 11**	3.6	4000	326.5	0.6	1:22517.4	1:42.9	1:42.9	2.6	0.04
**p 12**	2.4	4000	93.3	0.04	1:12.2	1:12.2	1:12.2	4000	0.6
**p 13**	2.3	93.3	93.3	93.3	1:12.2	1:1838.8	1:12.2	1142.9	26.5

All concentrations are given in µg/ml. Abbreviations: ATI, α-amylase/trypsin inhibitors; HMW-GS, high-molecular-weight glutenin subunits; eHWP, extensively hydrolyzed wheat proteins; sHWP, slightly hydrolyzed wheat proteins; sIgE, specific IgE.

Table S4. Concentrations of allergen test substances with highest median histamine release in patients with WALDA in the aBHRA.

Test substance in aBHRA	Concentration with highest median histamine release	Median histamine release (ng/ml)
Gluten	4000 µg/ml	25
HMW-GS	26.5 µg/ml	37.7
ATI	93.3 µg/ml	18.4
Alcohol-free wheat beer	1:12.2	31
eHWP	1:42.9	16.6
sHWP	1:42.9	29
Rye secalins	0.6 µg/ml	24.3
Rye gluten	1142.9 µg/ml	17.9

Table S5. Concentrations of allergen test substances with highest median histamine release in patients with WALDA in the pBHRA.

Test substance in pBHRA	Concentration with highest median histamine release	Median histamine release (ng/ml exceeding the negative control)
Gluten	3600 μg/ml	42.1
HMW-GS	3600 μg/ml	48.8
ATI	93.3 μg/ml	8.2
Alcohol-free wheat beer	1:12.2	36.1
eHWP	1:12.2	17.6
sHWP	1:12.2	18.3
Rye secalins	84 μg/ml	6.8

Table S6. Overview of the minimum concentration of test substances eliciting positive responses in WALDA patients.

In vitro test	Test substance	Minimum concentration
**BAT** %CD63+ >5%	Gluten	<800 μg/ml
	HMW-GS	<800 μg/ml
	ATI	80 μg/ml
	Wheat beer	<1:100
	eHWP	<1:50
	sHWP	<1:50
	Rye gluten	<800 μg/ml
	Rye secalins	<800 μg/ml
**aBHRA** histamine release >10 ng/ml	Gluten	<0.004 μg/ml
	HMW-GS	<0.004 μg/ml
	ATI	0.2 μg/ml
	Wheat beer	<1:964707
	eHWP	<1:964707
	sHWP	<1:964707
	Rye gluten	<0.004 μg/ml
	Rye secalins	<0.004 μg/ml
**pBHRA** histamine release >10 ng/ml exceeding the negative control	Gluten	293 μg/ml
	HMW-GS	293 μg/ml
	ATI	293 μg/ml
	Wheat beer	1:525.2
	eHWP	1:12.2
	sHWP	1:150.1
	Rye secalins	0.2 μg/ml

BAT, basophil activation test; aBHRA, active basophil histamine release assay; pBHRA, passive basophil histamine release assay; ATI, α-amylase/trypsin inhibitors; HMW-GS, high-molecular-weight glutenin subunits; eHWP, extensively hydrolyzed wheat proteins; sHWP, slightly hydrolyzed wheat proteins.
